# Loss of runt-related transcription factor 3 expression leads hepatocellular carcinoma cells to escape apoptosis

**DOI:** 10.1186/1471-2407-11-3

**Published:** 2011-01-04

**Authors:** Yutaka Nakanishi, Hidenori Shiraha, Shin-ichi Nishina, Shigetomi Tanaka, Minoru Matsubara, Shigeru Horiguchi, Masaya Iwamuro, Nobuyuki Takaoka, Masayuki Uemura, Kenji Kuwaki, Hiroaki Hagihara, Junichi Toshimori, Hideki Ohnishi, Akinobu Takaki, Shinichiro Nakamura, Yoshiyuki Kobayashi, Kazuhiro Nouso, Takahito Yagi, Kazuhide Yamamoto

**Affiliations:** 1 Department of Gastroenterology and Hepatology, Okayama University Graduate School of Medicine and Dentistry, 2-5-1 Shikata-cho, Okayama 700-8558, Japan; 2 Department of Molecular Hepatology, Okayama University Graduate School of Medicine and Dentistry, 2-5-1 Shikata-cho, Okayama 700-8558, Japan; 3 Department of Gastroenterological Surgery, Transplant, and Surgical Oncology, Okayama University Graduate School of Medicine and Dentistry, 2-5-1 Shikata-cho, Okayama 700-8558, Japan

## Abstract

**Background:**

Runt-related transcription factor 3 (RUNX3) is known as a tumor suppressor gene for gastric cancer and other cancers, this gene may be involved in the development of hepatocellular carcinoma (HCC).

**Methods:**

RUNX3 expression was analyzed by immunoblot and immunohistochemistry in HCC cells and tissues, respectively. Hep3B cells, lacking endogenous RUNX3, were introduced with RUNX3 constructs. Cell proliferation was measured using the MTT assay and apoptosis was evaluated using DAPI staining. Apoptosis signaling was assessed by immunoblot analysis.

**Results:**

RUNX3 protein expression was frequently inactivated in the HCC cell lines (91%) and tissues (90%). RUNX3 expression inhibited 90 ± 8% of cell growth at 72 h in serum starved Hep3B cells. Forty-eight hour serum starvation-induced apoptosis and the percentage of apoptotic cells reached 31 ± 4% and 4 ± 1% in RUNX3-expressing Hep3B and control cells, respectively. Apoptotic activity was increased by Bim expression and caspase-3 and caspase-9 activation.

**Conclusion:**

RUNX3 expression enhanced serum starvation-induced apoptosis in HCC cell lines. RUNX3 is deleted or weakly expressed in HCC, which leads to tumorigenesis by escaping apoptosis.

## Background

Hepatocellular carcinoma (HCC)^1 ^is the sixth most common cancer and responsible for more than half a million deaths worldwide each year [1-3]. Although most HCC cases occur in East Asia and Middle and West Africa, its incidence in some developed countries is increasing [1,4]. In most cases, HCC is fatal because of an incomplete understanding of the pathogenic mechanisms and inadequacies of early detection [1,5].

The activation of proto-oncogenes plays a major role in the development of HCC [1,6-8], and a number of tumor suppressor genes may be associated with the development and progression of HCC [1,9-12]. Although several cancer-related genes are altered in HCC, the frequency of alterations for each individual gene is relatively low. In HCC, the alteration of tumor suppressor genes seems to be more important than that of oncogenes. Established genetic events include the loss of an allele, mutation, or promoter methylation [13-16]. A higher loss of heterozygosity (LOH) frequency was detected at several loci on chromosomes 8p23, 4q22-24, 4q35, 17p13, 16q23-24, 6q27, 1p36, and 9p12-14, suggesting the presence of important tumor suppressor genes at these loci [17]. However, there is little understanding of the several key pathways and the genes involved in these pathways.

Runt-related transcription factor 3 (RUNX3), located on chromosome 1p36, is correlated with tumorigenesis and gastric cancer progression [18,19]. RUNX3 acts as an apoptotic factor, downstream of transforming growth factor-β (TGF-β), and as a cell differentiation mediator in intestinal metaplasia of gastric mucosa [19-21]. In gastric cancer cell lines, RUNX3-induced apoptosis depends on Bim expression [22]. RUNX3 protein expression is decreased about 45-60% in human gastric cancer [21] and has been detected in some human malignancies such as those of the colon, lung, pancreas, and bile duct [23-26]. RUNX3 gene expression decreased in 30-80% of HCCs due to LOH and methylation of its promoter [27,28]. The loss or decrease of RUNX3 expression in HCC tissue has been recently reported [29], but the precise function of RUNX3 in HCC needs to be elucidated.

## Methods

### Cell lines and cell culture

The HCC cell lines HepG2, Hep3B, PLC/PRF/5 (PLC), and SK-Hep1 were obtained from the American Type Culture Collection (Manassas, VA), and the Huh1, Huh7, JHH1, JHH2, JHH4, HLE, and HLF cell lines were obtained from the Health Science Research Resources Bank (Osaka, Japan). Normal human hepatocytes were obtained from Sanko Junyaku Co. Ltd. (Tokyo, Japan). JHH2 and normal human hepatocytes were cultured in William's medium E (Invitrogen, Carlsbad, CA). Other cell lines were maintained in Dulbecco's modified Eagle's medium (Invitrogen). Media were supplemented with 10% heat-inactivated fetal bovine serum (FBS) (Sigma, St. Louis, MO), 1% nonessential amino acids (Sigma), 1% sodium pyruvate (Sigma), and 1% penicillin/streptomycin solution (Sigma). Cells were cultured at 37°C in a humidified atmosphere of 5% CO_2 _and 95% air. Quiescence was carried out under restricted serum conditions with 0.1% dialyzed FBS for the indicated time periods.

### RNA preparation and reverse transcriptase-polymerase chain reaction

Total RNA was isolated from cells using Trizol™ reagent (Invitrogen). Reverse transcription was performed using random primers and ReverTra Ace™ (Toyobo, Osaka, Japan) reverse transcriptase (RT). Ps-CA and Ps-CB, previously published primer set for RUNX3, were utilized [21]. For each polymerase chain reaction (PCR), 20 μl (total volume) of reaction mixture contained 0.1 μg template DNA, 4 pmol each of the forward and reverse primers, 2 μl deoxynucleoside triphosphates (200 mM each), 1 U pfu Turbo™ DNA polymerase (Stratagene, La Jolla, CA), and 2 μl of 10× pfu reaction buffer. PCR amplification was conducted on an iCycler™ (Bio-Rad, Hercules, CA) with the following cycle conditions: cycle 1, 95°C for 2 min; cycles 2-30, 95°C for 30 s, 58°C for 30 s, and 72°C for 120 s, with a final elongation step of 72°C for 10 min.

### Immunoblot analysis

Cells were plated onto 6-well tissue culture plastic dishes and grown to confluence. After cultivating the cells under the indicated conditions, they were washed twice with cold phosphate-buffered saline (PBS) and lysed in 150 μl of sample buffer (100 mM Tris-HCl, pH 6.8, 10% glycerol, 4% sodium dodecyl sulfate [SDS], 1% bromophenol blue, 10% β-mercaptoethanol). The samples were resolved by SDS-polyacrylamide gel electrophoresis (PAGE) and transferred to Immobilon-P™ polyvinylidene difluoride membranes (Millipore Corporation, Bedford, MA), which were blocked using Tris-buffered saline with Tween-20 (TBS-T) (Sigma) containing 5% bovine serum albumin for 1 h. The membranes were incubated with antibodies against RUNX3 (R3-G54; Abcam, Cambridge, MA), poly-histidine (His) (Roche Diagnostics, Basel, Switzerland), Bax, Bcl-2, Bim, cleaved caspase-3 and -9 (Cell Signaling Technology, Beverley, MA), and β-actin (Sigma) overnight at 4°C. We washed the membranes three times with TBS-T and probed with horseradish peroxidase-conjugated secondary antibodies before developing them using an ECL Western blotting detection system (Amersham Biosciences, Piscataway, NJ) by enhanced chemiluminescence.

### HCC tissue and immunohistochemistry

Thirty-one patients including 24 men with age ranging from 18 to 71 years (average age, 58 years) and 7 women with age ranging from 59 to 67 years (average age, 63 years) at the time of hepatic resection were included in this study. HCC tissues along with adjacent liver tissues were used for analysis. As per the institutional guidelines, we obtained informed consent from all donors of liver tissue samples, and the study was approved by the Research Ethics Committee of Okayama University.

Immunohistochemistry was performed on formalin-fixed paraffin sections that were dewaxed and dehydrated. After rehydration, endogenous peroxidase activity was blocked for 30 min in a methanol solution containing 0.3% hydrogen peroxide. After antigen retrieval in citrate buffer, the sections were blocked overnight at 4°C. The sections were probed with rabbit polyclonal antibody (ab49117; Abcam) followed by biotinylated anti-rabbit secondary antibody (Dako Japan, Tokyo, Japan). The signal was amplified by avidin-biotin complex formation and developed with diaminobenzidine followed by counterstaining with hematoxylin, after which the sections were dehydrated in alcohol and xylene, and mounted for observation. The sections were scored on a four-tier scale; 0, negative; 1, weak signal; 2, intermediate signal; and 3, strong signal [30]. All sections were scored independently by two observers (Y. K. and K. N.) without prior knowledge. All discrepancies in scoring were reviewed and a consensus was reached.

### RUNX3 cloning and transfection

We obtained human RUNX3 cDNA by PCR-based cloning from normal human hepatocytes (Sanko Junyaku). Briefly, cDNA was amplified by PCR using sense (5'-TATGCGTATTCCCGTAGA) and antisense (5'-CTCGAGGCGGCCGCTCAATGGTGATGGTGATGATGACCGGTACGGTAGGGCCGCCACAC; including the six-His tag) oligonucleotide primers with Pfu Turbo™ Hotstart DNA polymerase (Stratagene) and cloned into the PCR II TA cloning vector (Invitrogen). The size of the PCR product was ~1.2 kb. After confirmation by sequencing, RUNX3 cDNA was subcloned into pCEP4 (Stratagene), downstream from a cytomegalovirus promoter. The poly-His tag was replaced with green fluorescent protein (GFP) cDNA from pEGFP-C1 (Clontech, Palo Alto, CA). The human RUNX3 and/or chloramphenicol acetyltransferase (CAT) (control) constructs were transfected into Hep3B cells using FuGENE™6 transfection reagent (Roche), as per the manufacturer's instruction. Cells were selected in complete medium containing 250 μg/ml of hygromycin (Roche). Polyclonal lines consisting of more than 20 colonies were established. At least two independent stably transfected lines were established for each construct.

Transient RUNX3 expression was also conducted using FuGENE™6 in Hep3B, Huh7, HLE, and HLF cells. After transfection, the cells were cultured under serum starved condition for the indicated periods, if needed, and utilized for the following experiments.

### MTT assay

Cell proliferative activity was assessed with the 3-(4, 5-dimethylthiazol-2-yl)-2, 5-diphenyl tetrazolium bromide (MTT) assay. Briefly, cells were seeded at 2,000 cells/well in 96-well tissue culture plastic dishes and quiesced for 6 h with 0.1% dialyzed FBS. After 24-120 h of quiescence, the cells were cultured for the indicated periods with or without 10% FBS. At the end of the treatment, 10 μl of MTT (5 mg/ml in PBS) was added to each well, and the wells were incubated for an additional 2 h at 37°C. The purple-blue MTT formazan precipitate was dissolved in 200 μl of dimethyl sulfoxide (Sigma). The activity of the mitochondria, reflecting cellular growth and viability, was evaluated by measuring the optical density at 570 nm with a microplate reader (Bio-Rad).

### DAPI staining

Cells were plated at 50% confluence on glass chamber slides (Labtek II, Nalgen Nunc, Roskide, Denmark) and quiesced for 6 h with a media containing 0.1% dialyzed FBS. Then, they were treated with 10% FBS, 100 μM caspase inhibitor (caspase inhibitor IV, Calbiochem, Gibbstown, NJ), 1 nM transforming growth factor-α (TGF-α) (Peprotech Inc. Rocky Hill, NJ), 1 nM epidermal growth factor (EGF) (Peprotech), and/or 5 ng/ml platelet derived growth factor (PDGF)-BB (Peprotech). Chromosomal DNA was stained with 4', 6-diamidine-2'-phenylindole dihydrochloride (DAPI) (Dojindo, Kumamoto, Japan) according to the manufacturer's instructions. Briefly, treated cells were washed with PBS and stained with DAPI working solution (1 μg/ml in PBS) for 2 min. The percentage of cells with condensed chromatin and/or fragmented nuclei was established in 300-500 DAPI-stained cells examined under a fluorescence microscope (IX-70, Olympus, Tokyo, Japan).

### Flow cytometry analysis

Annexin V and propidium iodide (PI) staining was performed using an annexin V-fluorescein isothiocyanate (FITC) Apoptosis Detection kit (Medical & Biological Laboratories Co., Ltd., Nagoya, Japan) to measure apoptosis. Cells were cultured in 10-cm tissue culture plates and quiesced for 6 h with a media containing 0.1% dialyzed FBS. Cells were cultured in medium with or without 10% FBS for 24 h. Then, they were washed twice with PBS, collected, and re-suspended in 85 μl of 1× annexin V-FITC binding buffer. Five microliters of annexin V-FITC conjugate and 10 ml of PI buffer were added, and the cells were incubated at room temperature for 15 min in the dark. After adding 400 μl of 1× annexin V-FITC binding buffer, cells were analyzed using a flow cytometer (FACS Calibur; Becton Dickinson, Franklin Lakes, NJ).

### Gene silencing of Bim with small interfering RNA

RUNX3-expressing Hep3B cells were transfected with either scrambled negative control small interfering RNA (siRNA) or Bim siRNA (Applied Biosystems, Foster City, CA). siRNAs were transfected into cells using RNAiFect™ transfection reagent (Qiagen, Hilden, Germany). Cells were incubated with scrambled negative control siRNA or Bim siRNA for 24 h before 48 h of serum starvation. The MTT assay and DAPI staining for detecting apoptosis were performed as described above.

## Results

### Loss of RUNX3 expression in HCC cell lines and human HCC tissues

**Figure 1:**
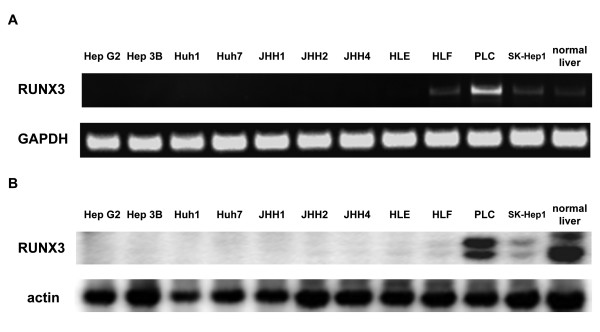
**RUNX3 mRNA (A) and protein (B) expression in HCC cell lines**. (A) RUNX3 and GAPDH mRNA expression levels were determined by RT-PCR. Shown here are representative gels from three independent experiments. (B) RUNX3 and α-actin protein expression was analyzed by immunoblot using the anti-RUNX3 antibody. Shown here are representative blots from more than three independent experiments.

A decreased level or absence of RUNX3 mRNA expression was observed in 10 of 11 HCC cell lines (Figure 1A). RUNX3 mRNA was undetectable in eight cell lines (HepG2, Hep3B, Huh1, Huh7, JHH1, JHH2, JHH4, and HLE). In HLF and SK-Hep1 cells, RUNX3 mRNA was significantly underexpressed (Figure 1A). Normal human hepatocytes expressed RUNX3 mRNA. Sequence analysis was performed in HLF, PLC, and SK-Hep1 cells, and no mutation was detected. In accordance with the mRNA analysis, RUNX3 protein expression was undetectable in the HepG2, Hep3B, JHH1, JHH2, JHH4, HLE, and HLF cell lines, while the RUNX3 protein was expressed in HLF, PLC, and SK-Hep1 cells (Figure 1B). The RUNX3 protein was significantly underexpressed in HLF and SK-Hep1 cells.

**Figure 2:**
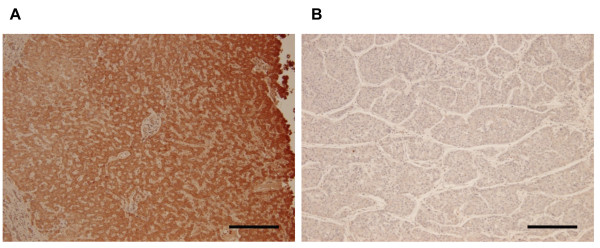
**Immunohistochemical staining of RUNX3 in human liver tissues**. (A) nontumor liver tissue with a protein score of 3 (B) HCC tissue with a protein score of 0. Bar = 100 μm.

RUNX3 protein expression in human HCC tissue was compared to that in the corresponding tumor-free resection margins using immunohistochemical analysis (Figure 2). Twenty eight (~90%) of these pairs showed a negative or weak signal for RUNX3 expression in HCC tissue, but showed RUNX3 protein expression in tumor-free resection margins (Table 1). In the remaining three pairs, a weak RUNX3 expression signal was detected in the tumor-free resection margins; thus, no negative RUNX3 signal was detected in the tumor-free resection margins.

**Table 1:** RUNX3 expression in HCC samples (n = 31) and the corresponding tumor-free resection margins

RUNX3 protein expression score	HCC samples (n = 31)	Tumor-free sections (n = 31)
0 (negative signal)	13 (41.9%)	0
1 (weak signal)	15 (48.4%)	3 (9.7%)
2 (intermediate signal)	3 (9.7%)	16 (51.6%)
3 (strong signal)	0	12 (38.7%)

### Ectopic RUNX3 protein expression in Hep3B cells

**Figure 3:**
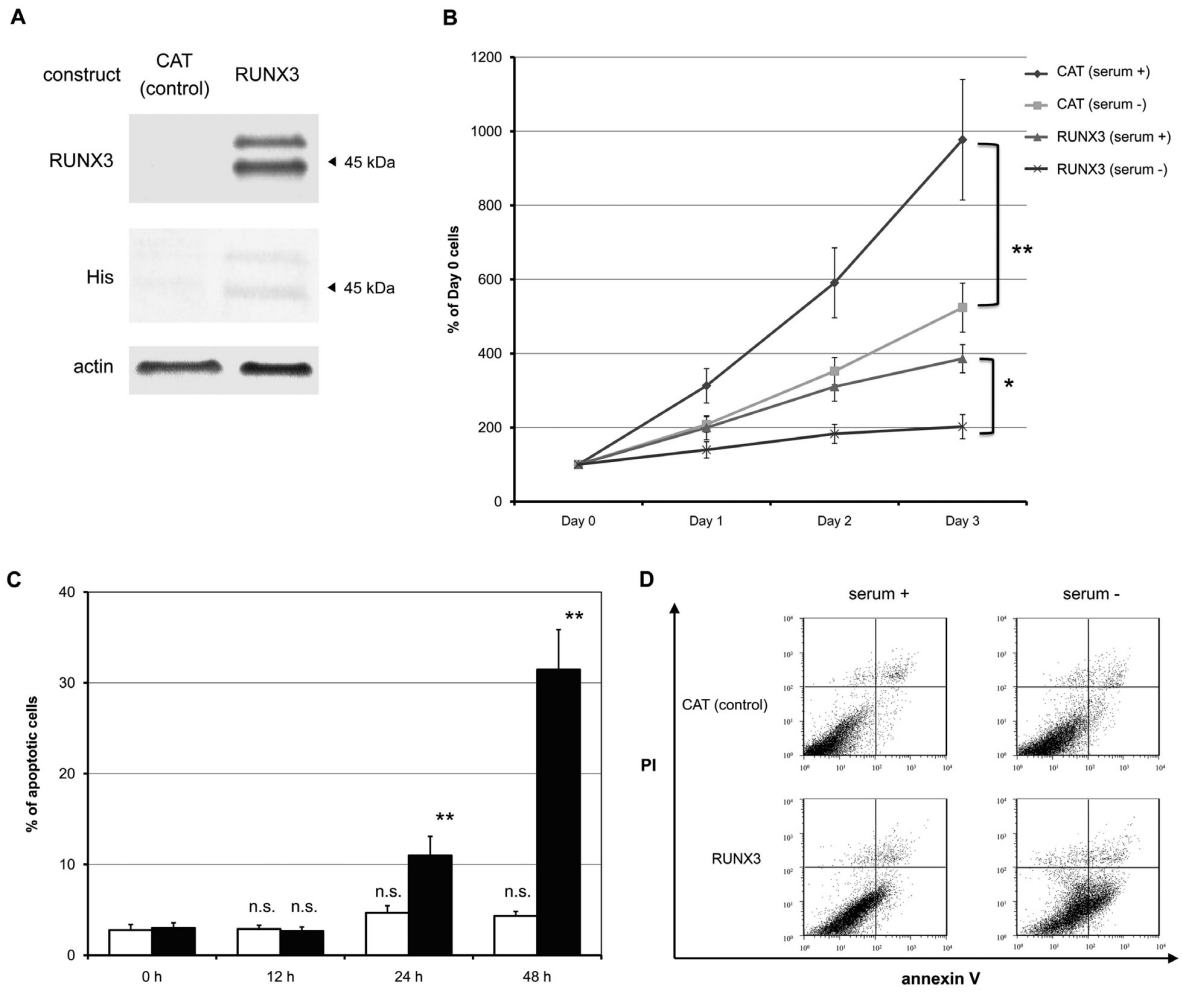
**RUNX3 expression in Hep3B cells**. CAT (control) and RUNX3 constructs were introduced into Hep3B cells. Polyclonal stably expressing cell lines were selected in the presence of hygromycin. (A) Immunoblot analysis RUNX3 protein expression was analyzed by immunoblot using anti-RUNX3 and anti-His antibodies. Shown here are representative blots from more than three independent experiments. (B) Effect of RUNX3 expression on cell growth Cell proliferative activities were measured by the MTT assay. All results are expressed as ratios to cell number at day 0. Data represent the mean ± S.E. of more than three independent experiments, each with four replicates. *, *P *< 0.05; **, *P *< 0.01 (vs. data at 0 h); Student's *t-*test. (C) Apoptosis determined by DAPI staining Cells were cultured in medium without FBS for the indicated periods. The cells were stained with DAPI, and the percentage of apoptotic cells was examined by fluorescence microscopy (CAT; white bars, RUNX3; black bars). Data represent the mean ± S.E. of more than five independent experiments, each with triplicates. n.s., not significant; **, *P *< 0.01 (vs. data at 0 h); Student's *t*-test. (D) Flow cytometry analysis Cells were cultured in medium with or without 10% FBS for 24 h. They were harvested and washed in PBS without Ca^2+^/Mg^2+^, and stained using the Mebcyto™ apoptosis detection kit. The cells were analyzed with a Becton Dickinson FACS Calibur flow cytometer. Shown here are representative plots from more than three independent experiments.

To assess whether RUNX3 protein expression affected cell survival in the HCC cell lines, a RUNX3 construct was introduced into RUNX3-negative Hep3B cells (Figure 3A). Overall, the clones were expressed at similar levels in all cells, as determined by immunocytochemical analysis (data not shown). RUNX3-expressing Hep3B cells grew slightly slower than normal Hep3B cells in the presence of FBS.

### RUNX3 expression inhibited cell growth under serum starvation

RUNX3 has been reported to induce apoptosis in a gastric cancer cell study [21]. The MTT assay was performed to determine whether RUNX3 expression influenced cell growth. RUNX3-expressing Hep3B cells grew slightly slower than CAT-transfected Hep3B cells in the presence of FBS, whereas the growth of RUNX3-expressing Hep3B cells was markedly suppressed in the absence of FBS; growth inhibition could be observed as early as 24 h, and reached 70 ± 12% and 90 ± 8% at 48 and 72 h, respectively (Figure 3B). The inhibition levels were over 4 times than those found in the condition with 10% FBS. This effect was confirmed with GFP-tagged RUNX3-expressing Hep3B cells (70 ± 11% growth inhibition at 72 h).

### RUNX3 expression induced apoptosis under serum starvation

The effect of RUNX3 expression on cell survival and the cell cycle with and without FBS was assessed to investigate whether the elicited growth suppression in RUNX3-expressing cells under serum starved conditions was due to an increase in cell death or due to cell cycle inhibition, or both. DAPI staining demonstrated that serum starvation induced apoptosis in RUNX3-expressing Hep3B cells (31 ± 4%) but not in CAT-transfected Hep3B cells (4 ± 1%) in the absence of FBS (Figure 3C). Flow cytometry analysis with annexin V antibody was also performed. RUNX3-expressing Hep3B cells showed a significant increase in a pre-apoptosis population (Annexin V+ PI-) after 24 h of serum starvation compared with CAT-transfected Hep3B cells (Figure 3D).

### RUNX3-induced apoptosis through the Bim-caspase pathway

**Figure 4:**
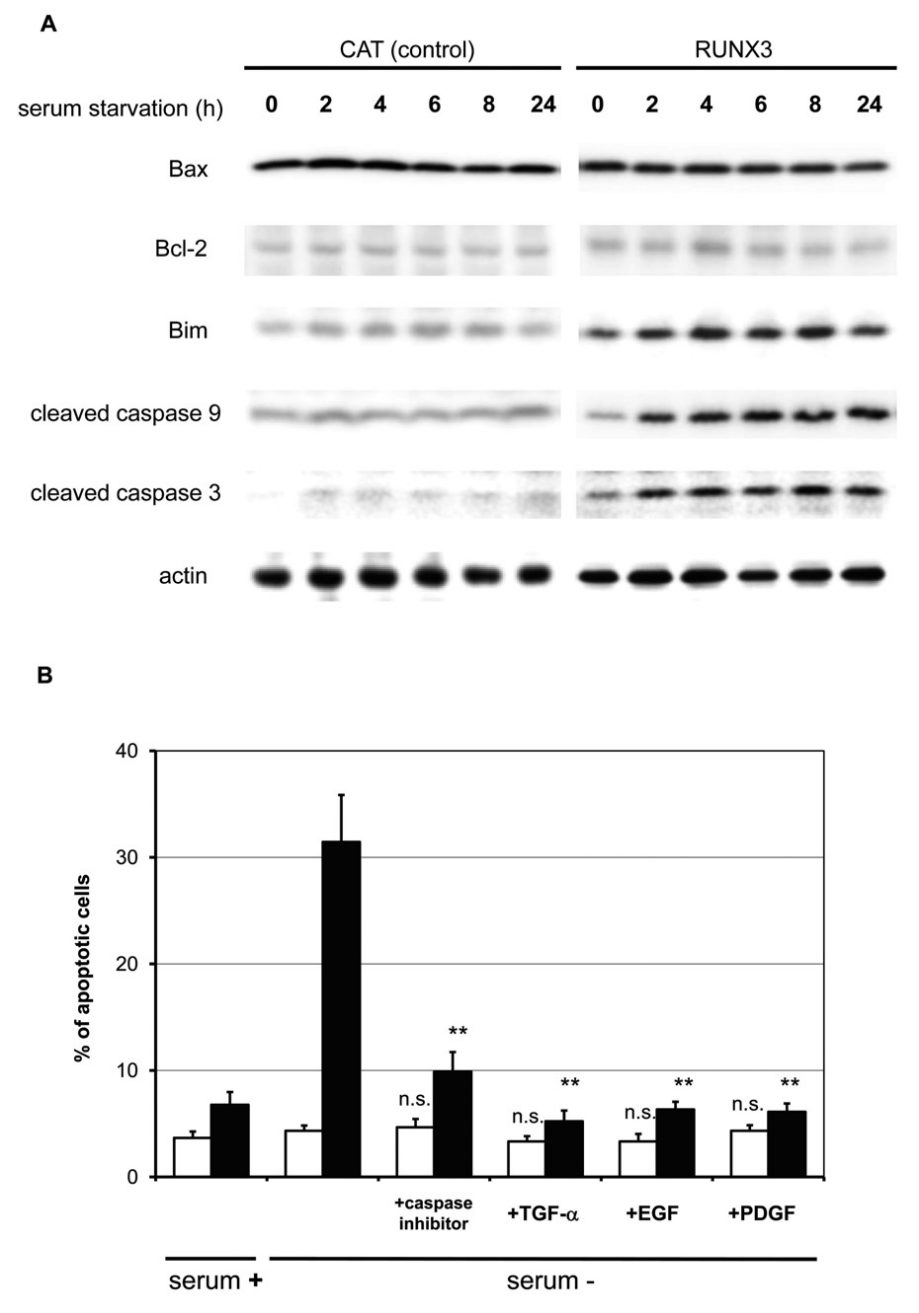
**Effect of RUNX3 on apoptosis-related signaling molecules**. (A) Immunoblot analysis Equal amounts of cell lysates were size-fractionated with 10% SDS-PAGE and immunoblotted with antibodies against Bax, Bcl-2, Bim, cleaved caspase-9, cleaved caspase-3, and actin. Shown here are representative blots from at least three independent experiments. (B) Apoptosis determined by DAPI staining Cells were cultured in media with or without 10% FBS, caspase inhibitor, and/or TGF-α for 48 h. Cells were stained with DAPI, and the percentage of apoptotic cells was determined under a fluorescent microscope (CAT; white bars, RUNX3; black bars). Data represent the mean ± S.E. of more than five independent experiments, each with triplicates. n.s., not significant; *P *> 0.05; **, *P *< 0.01 (vs. data with no serum); Student's *t*-test.

Because a RUNX3-induced apoptotic pathway has been described previously, the effect of altering RUNX3 expression was investigated. Bim protein expression was enhanced by serum starvation in RUNX3-expressing Hep3B cells but not in control cells (Figure 4A). Activated apoptosis executors, caspase-9 and -3, were found in serum starved RUNX3-expressing Hep3B cells. Expression of the Bim attenuators, Bax and Bcl-2, was not affected by serum starvation. These results imply that Bim plays a major role in serum starvation-induced apoptosis in RUNX3-expressing cells.

Serum starvation-induced apoptosis was abrogated by an apoptosis inhibitor (Figure 4B). Various growth factors were employed to determine whether serum starvation-induced apoptosis was caused by the absence of a growth factor-induced survival signal. As a result, TGF-α, EGF, and PDGF abrogated serum starvation-induced apoptosis in RUNX3-expressing Hep3B cells (Figure 4B).

### siRNA against Bim reduced serum starvation-induced apoptosis in RUNX3-expressing Hep3B cells

**Figure 5:**
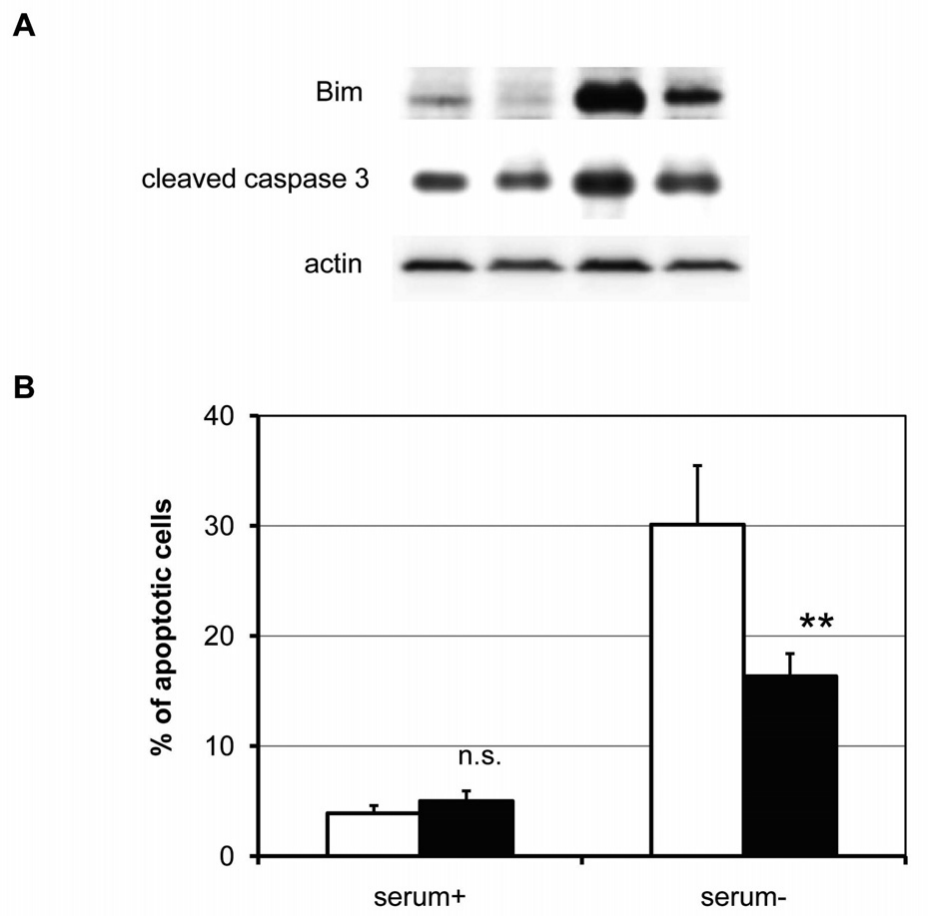
**Effect of Bim siRNA gene silencing on cell growth inhibition and apoptosis**. After treatment with Bim siRNA duplexes, an immunoblot analysis (A) and DAPI apoptosis detection assay (B) were performed. Shown here are representative blots from more than three independent experiments. All results are expressed as ratios to control siRNA-treated cells (control siRNA, white bars; Bim siRNA, black bars). Data are mean ± S.E. from at least three independent experiments, each performed in triplicate. Statistical analysis was performed with the Student's *t*-test. n.s., not significant; **, *P *< 0.01 (vs. control siRNA-treated cells).

siRNA against Bim was used to knockdown Bim expression in Hep3B cells (Figure 5A). The expression level of cleaved caspase-3, decreased in Bim siRNA-treated cells (Figure 5A). Bim siRNA inhibited serum starvation-induced apoptosis by 46 ± 7% in RUNX3-expressing Hep3B cells (Figure 5B).

### Transient ectopic RUNX3 expression in various HCC cell lines

**Figure 6:**
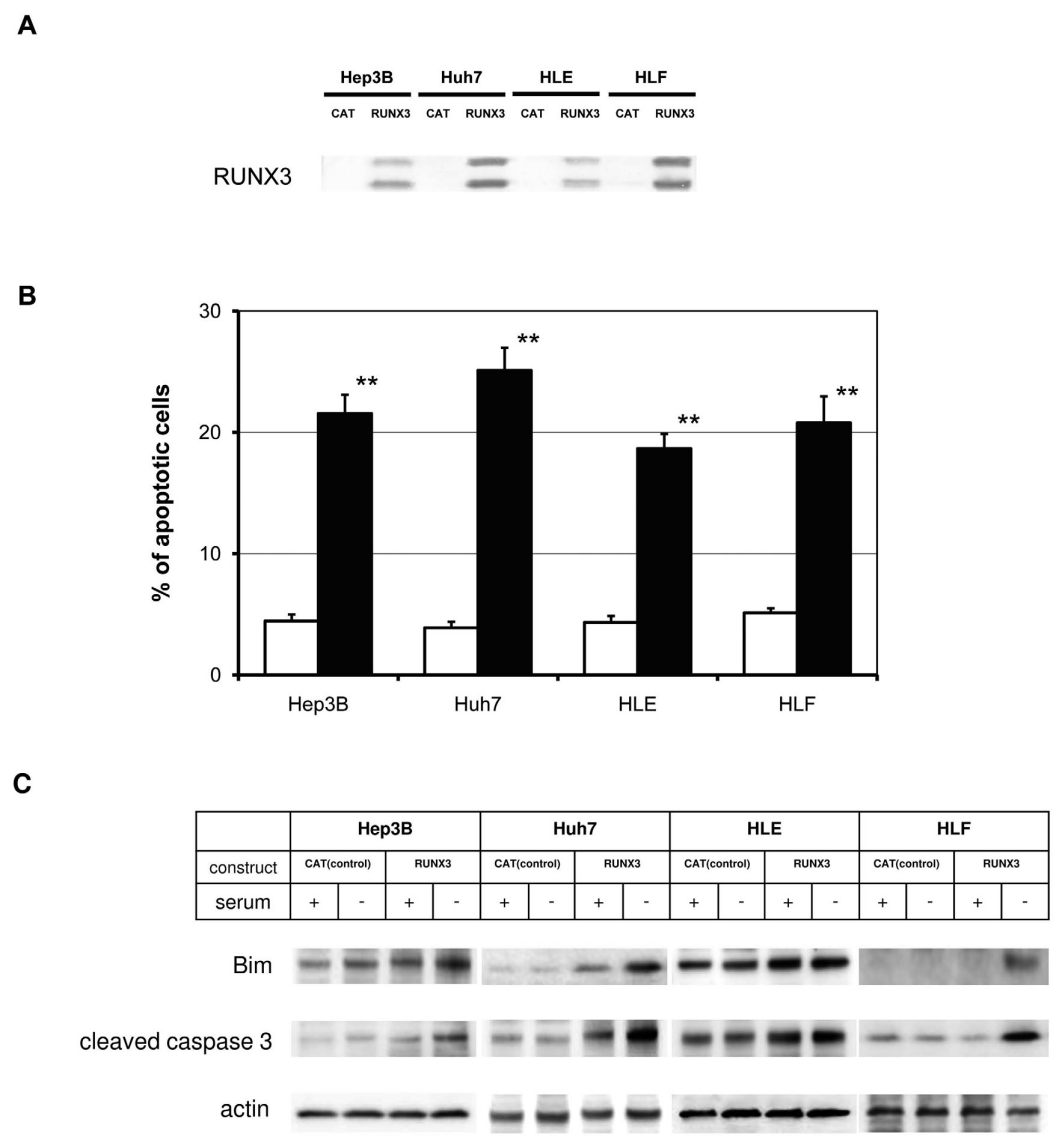
**Effect of transient ectopic RUNX3 expression**. CAT (control) and RUNX3 constructs were introduced into HCC cell lines. After a 48-h incubation period, an immunoblot analysis for RUNX3 expression (A), a DAPI apoptosis detection assay (B), and an immunoblot analysis for Bim and caspase (C) were performed. Shown here are representative blots from more than three independent experiments. All results are expressed as ratios to control CAT-transfected cells (CAT; white bars, RUNX3; black bars). Data represent the mean ± S.E. of more than five independent experiments, each with triplicates. **, *P *< 0.01 (vs. data at 0 h); Student's *t*-test.

RUNX3 was transiently expressed in various HCC cell lines, including Hep3B, Huh7, HLE, and HLF, introducing a RUNX3 construct-induced RUNX3 protein expression (Figure 6A). Transient RUNX3-expressing cells also showed growth inhibition after 48 h of serum starvation; the inhibition was 50 ± 10%, 46 ± 11%, 60 ± 8%, and 52 ± 9% in Hep3B, Huh7, HLE, and HLF cells, respectively. The RUNX3-expressing HCC cell lines demonstrated enhanced serum starvation-induced apoptosis; the percentage of apoptotic cells determined by DAPI staining was 21 ± 2%, 25 ± 2%, 19 ± 1%, and 20 ± 2% in Hep3B, Huh7, HLE, and HLF cells, respectively (Figure 6B). Serum starvation-induced Bim expression and caspase-3 cleavage were also confirmed in RUNX3-expressing Hep3B, Huh7, HLE, and HLF cells (Figure 6C).

## Discussion

The results of the present study demonstrated that RUNX3 is a tumor suppressor gene for HCC. A significant down-regulation of RUNX3 was observed in a high percentage of human HCC cell lines (91%) and tissues (90%) (Figures 1, 2, and Table 1). RUNX3 has been described as a gastric cancer tumor suppressor [21]. In many cancer types, deletion of the RUNX3 locus and reduction of its expression by promoter hypermethylation has been reported [23-26]. However, little is known about the role of RUNX3 in HCC tumor suppression. We hypothesized that loss of RUNX3 expression contributes the development of HCC by escaping apoptosis. The results of the present study provide clear evidence that RUNX3 elicits serum starvation-induced apoptosis in HCC cells by activating the Bim-caspase pathway.

Stable expression of RUNX3 protein was established in Hep3B cells (Figure 3A), and they showed apoptosis under serum starved conditions (Figure 3B). This effect was reproducible in the Hep3B, Huh7, HLE, and HLF HCC cell lines transiently expressing RUNX3. The inhibition of cell growth in transient RUNX3-expressing cells was generally lower than that in stable RUNX3-expressing Hep3B cells, probably due to low transfection efficiency.

Serum starvation-induced apoptosis is caused by caspase activation in ectopic RUNX3-expressing Hep3B cells (Figures 3C and 3D). To explore the signaling molecule responsible for apoptosis, Bim protein expression was induced in serum starved RUNX3-expressing Hep3B cells (Figure 4A). This is the first report demonstrating that RUNX3 enhances Bim expression under serum starved conditions in HCC cells, which appears to be consistent with the important role of Bim in previous studies on other types of cells. Bim expression was induced by the cooperation of RUNX3 and TGF-β in a study of gastric epithelial cells [21,31]. Bim protein also plays an important role in cell death [32]. Bim induces sequential activation of caspase-9 and -3 [32]. The potency of Bim as a cell death inducer is attenuated by Bax and Bcl-2 subfamily proteins [33]. The expression of Bax and Bcl was not affected by RUNX3 expression (Figure 4A). The expression of Bad (data not shown), a Bcl-2 antagonist known as a serum starvation-induced apoptosis initiator [34], increased with serum starvation but was not attenuated by RUNX3 expression (Figure 4A). Bim siRNA was used to evaluate whether Bim expression regulates serum starvation-induced apoptosis in RUNX3-expressing cells. As a result, Bim siRNA successfully knocked down Bim expression in RUNX3-expressing Hep3B cells (Figure 5A). Knockdown of Bim expression abrogated serum starvation-induced apoptosis in RUNX3-expressing Hep3B cells (Figure 5B). Consequently, RUNX3 expression enhanced serum starvation-induced apoptosis through the Bim-caspase pathway in Hep3B cells. This effect was reproducible in the Huh7, HLE, and HLF HCC cell lines transiently expressing RUNX3 (Figure 6).

Serum starvation triggered apoptosis in RUNX3-expressing HCC cells. As this leads to the question of how serum prevents apoptosis in RUNX3-expressing cells, RUNX3-expressing Hep3B cells were treated with TGF-α, EGF, or PDGF (Figure 4C). These growth factors reduced apoptosis in RUNX3-expressing Hep3B cells by activating the PI3/Akt signaling pathway (data not shown), which is consistent with a previous report [34].

RUNX3 induces apoptosis in the presence of TGF-β [21]. In a study of gastric epithelial cells, RUNX3 enhanced Bim expression during TGF-β-induced apoptosis [21,31]. In a study of a gastric and esophageal cancer cell lines, RUNX3 expression made cancer cells sensitive to TGF-β-induced apoptosis [21,35-38]. These reports suggest that TGF-β is required for RUNX3-related apoptosis. In the present study, ectopic RUNX3 expression enhanced serum starvation-induced apoptosis in the absence of TGF-β. This discrepancy may be explained by the autocrine action of TGF-β in Hep3B cells, which have an intact TGF-β signaling pathway [39]. Furthermore, some HCC cell lines, including Hep3B, produce TGF-β [40]. Further study is required to establish whether TGF-β is involved in the enhanced apoptosis of HCC.

It has been reported that p53, Rb, p16, phosphatase, and tensin homolog (PTEN) are altered in HCC. The p53 gene is the most extensively studied gene of solid tumors. Alteration of this gene occurs at a relative low frequency (28-42%) in HCC compared to other solid tumors [11,17,41,42]. The Rb gene is another well-studied tumor suppressor gene in HCC and other solid tumors. Rb mutations are found in only 15% of HCCs [42]. The LOH of chromosome 13q, where Rb gene is located, is more frequent in HCC (25-48%) [43,44]. The p16 gene, also known as the cyclin-dependent kinase inhibitor 2A gene, regulates the Rb pathway and is found in 64% of HCCs [9]. PTEN negatively regulates the PI3K/Akt signaling pathway, which is involved in the regulation of cell survival [45]. Alteration of PTEN was found in ~40% of HCCs [10]. The frequency of alteration of each individual gene was relatively low, while RUNX3 expression was frequently down-regulated in both human HCC cell lines (91%) and tissues (90%).

Alterations in some tumor suppressor genes are due to LOH in HCC [17]. Similar to other tumor suppressor genes, some of the alterations in RUNX3 are due to the LOH of chromosome 1p36, where RUNX3 is located. Perhaps another mechanism for RUNX3 down-regulation is hypermethylation of the RUNX3 promoter region [13-16]. In a previous report, 30-40% of HCCs showed LOH of the RUNX3 gene and 40-80% showed promoter hypermethylation [28]. In agreement with these reports, RUNX3 down-regulation was detected in ~90% of HCC tissue specimens.

## Conclusions

RUNX3 expression elicits serum starvation-induced apoptosis in HCC cells via the Bim-caspase pathway. Because RUNX3 expression is generally suppressed in HCC cell lines and tissues, loss of RUNX3 expression leads to tumorigenesis by escaping apoptosis.

## Competing interests

The authors declare that they have no competing interests.

## Authors' contributions

HS conceived the design and drafted the manuscript. YN performed experiments. NT, ST, SN, MU, MM, MI and AT helped performing experiments for YN. SN, YK, KN, KK, HH, JT, HO and TY contributed for the collection of HCC tissues. YN performed immunohistochemical study. KY provides financial supports and participates in the discussion of the results. All authors read and approved the final manusctipt.

## Pre-publication history

The pre-publication history for this paper can be accessed here:

http://www.biomedcentral.com/1471-2407/11/3/prepub
